# A novel short-course, low-intensity blood-flow-restricted exercise (BFRE) regimen to study satellite cell function in critical illness survivors with sustained muscle atrophy following intensive care unit-acquired weakness (ICUAW)

**DOI:** 10.3389/fphys.2025.1553471

**Published:** 2025-06-18

**Authors:** Sunita Mathur, Nathalia P. S. Maia, Manoela De Paula Ferreira, Christian Martin, Christina Doherty, Judy Correa, Caterina Di Ciano-Oliveira, Pamela J. Plant, Jane Batt

**Affiliations:** ^1^ Department of Physical Therapy, Temerty Faculty of Medicine, University of Toronto, Toronto, ON, Canada; ^2^ Keenan Research Center for Biomedical Science, St Michael’s Hospital Unity Health Toronto, Toronto, ON, Canada; ^3^ Department of Medicine, Temerty Faculty of Medicine, University of Toronto, Toronto, ON, Canada

**Keywords:** quadriceps, critical illness myopathy, satellite cell, resistance exercise, MuRF1, myostatin, blood-flow-restricted exercise, intensive care unit acquired weakness

## Abstract

**Introduction:**

ICU-acquired weakness (ICUAW) develops in critically ill patients and can persist after hospital discharge, resulting in physical disability. Decreased satellite cell content is reported in the atrophic muscle of critical illness survivors, suggesting that the sustained muscle wasting results from satellite cell dysfunction and impaired muscle regeneration. Intense resistance exercise stimulates satellite cell proliferation and can be used to study the satellite cell role in persisting muscle atrophy following ICU discharge; however, the intensity of exercise required can be intolerable for older or frail ICU survivors. This study tested the capacity of a novel low-intensity, short-duration blood-flow-restricted exercise (BFRE) regimen, designed to accommodate the physical exercise limitations of critical illness survivors, to stimulate the satellite cell.

**Methods:**

Eight healthy controls (five men, three women, ages 20–64 years) underwent five consecutive daily sessions of quadriceps BFRE consisting of eight sets of eight knee extensions at 30% isometric peak torque followed by imaging and vastus lateralis (VL) biopsy to determine the quadriceps’ size, strength, VL satellite cell content, and transcript expression levels of regulators of muscle proteolysis, autophagy, and myogenic regulatory factors pre- and post-BFRE training. The BFRE regimen was piloted in three ICUAW survivors (ages 54–62 years) 5 years post-ICU discharge.

**Results:**

All study participants tolerated and completed the BFRE regimen. In controls, satellite cell content and MuRF1 transcript expression were significantly higher (1.53 ± 0.30- and 1.34 ± 0.31-fold difference, respectively) and myostatin transcript expression was significantly lower (0.58 ± 0.31-fold difference) in BFRE-trained versus untrained VL. Two survivors with low quadriceps mass compared to sex- and age-matched population-based norms and study controls showed no difference in satellite cell content in trained vs. untrained VL. In the survivor with quadriceps mass comparable to population norms and controls, satellite cell content was higher in the BFRE-trained versus untrained VL.

**Conclusion:**

This study demonstrates that training with a novel short-duration, low-intensity BFRE regimen results in higher satellite cell content in healthy muscle and can be completed by ICUAW survivors. Pilot data suggest that sustained satellite cell dysfunction may impede muscle mass reconstitution after ICU discharge.

## Introduction

Weakness develops in critically ill patients due to muscle wasting, impaired contractility, and peripheral nerve dysfunction—a phenomenon referred to as intensive care unit acquired weakness (ICUAW) (Batt et al., 2019). Muscle atrophy and weakness can persist indefinitely after ICU discharge, with as few as 50% of critical illness survivors returning to “normal physical function” depending on the cohort assessed (Intiso et al., 2022; Herridge et al., 2016). The trajectory of physical functional improvement is greatest during the first 6 months following ICU discharge, after which recovery slows and plateaus by 1 year (Herridge et al., 2016; Dos Santos et al., 2016). Patients with sustained muscle wasting and weakness post-ICU discharge experience physical disability that negatively impacts their quality of life and ability to return to work and live independently (Herridge et al., 2016). There are currently no therapies that universally prevent or reverse ICUAW (Batt et al., 2019; Lad et al., 2020; Batt and Mathur, 2017).

Research in both animal models and humans has delineated the cellular and molecular mechanisms that underpin ICUAW. In ICUs, loss of muscle mass and impaired contractility result from the combination of upregulated skeletal muscle proteolytic degradation, dysregulated autophagy, mitochondrial loss and dysfunction, and satellite cell loss (dos Santos et al., 2016; Puthucheary et al., 2013; Mankowski et al., 2022; Rocheteau et al., 2015; Banduseela et al., 2009; Ochala et al., 2011a; Ochala et al., 2011b; Norman et al., 2006; Aare et al., 2011; Aare et al., 2012; Ackermann et al., 2014). In contrast, the mechanisms responsible for the persistence of muscle wasting and weakness versus the recovery of muscle mass and strength after ICU discharge remain poorly understood. Animal models of ICUAW are unable to adequately recapitulate the long-term muscle sequelae for mechanistic study. Research in humans has shown that muscle satellite cell content is persistently decreased in critical illness survivors with sustained muscle atrophy and weakness following ICU discharge (dos Santos et al., 2016), while muscle proteolytic and autophagic cellular signaling and mitochondrial size and number are normalized. Satellite cells are muscle resident stem cells that proliferate and selectively differentiate to myocytes that fuse to form myofibers, regenerating injured muscle via hyperplastic growth (Kaczmarek et al., 2021; Snijders et al., 2015). ICU-induced satellite cell loss and/or dysfunction may causally contribute to the inability of patients to regain muscle mass and strength following ICU discharge.

Delineation of the cellular and molecular mechanisms responsible for sustained muscle wasting and weakness in critical illness survivors is essential to advancing therapeutic strategies for muscle recovery, and the satellite cell is a potential target. Intense, repetitive resistance exercise with high workloads exceeding 60%–70% of maximal strength stimulates satellite cell proliferation and muscle hyperplastic growth, as well as muscle hypertrophy via the regulation of proteolysis and protein synthesis (Kraemer et al., 2002; Kadi et al., 2004; Mackey et al., 2009). Such exercise can be utilized for the mechanistic study of persistent muscle wasting following ICU discharge. High-intensity resistance exercise, however, can be excessive and not achievable by individuals who are older, have chronic medical conditions, or are frail (Fragala et al., 2019; Gheno et al., 2012), such as some critical illness survivors. In contrast, blood flow restricted exercise (BFRE), which involves the application of a circumferential compressive band on the proximal portion of the limb to limit blood flow, stimulates satellite cell proliferation and muscle hypertrophy in response to much lower exercise loads (e.g., 20%–30% of maximal strength), although the satellite cell response is more variable and exercise duration-dependent (Gheno et al., 2012; Reina-Ruiz et al., 2022; Nielsen et al., 2012; Wernbom et al., 2013). The tolerability of BFRE in ICU survivors and its impact on the satellite cell and muscle regenerative and hypertrophic growth has not been assessed.

The purpose of this pilot study was to determine whether a novel BFRE protocol, designed to be of minimum intensity and duration so as to be achievable for ICU survivors, 1) retained the ability to stimulate satellite cell proliferation in healthy individuals and recapitulate the changes in molecular regulators of muscle mass reported in the literature for high-intensity resistance exercise and 2) is tolerated and can be completed by critical illness survivors. The BFRE regimen could thereby serve as a potential tool for future mechanistic study of sustained muscle atrophy following ICU discharge.

We report that all study participants completed the novel BFRE regimen. In the healthy control subjects, we show that the satellite cell content was significantly higher in the BFRE trained versus untrained muscle and that the differences observed in transcript levels of proteins that regulate proteolysis and muscle mass (MuRF1 [Muscle RING finger protein-1], Atrogin-1, myostatin), autophagy (Beclin-1, LC3 [Microtubule associated protein light chain 3]) and post-natal myogenesis (Myf5 [Myogenic factor 5], myogenin) (Peris-Moreno et al., 2021; Chen et al., 2021; Sebastian and Zorzano, 2020; Perry and Rudnicki, 2000) were consistent with the changes previously reported for high-intensity, long-duration resistance exercise protocols, thus demonstrating the regimen’s effectiveness as a study intervention. Although this study was not powered to assess the impact of the BFRE regimen on muscle satellite cell content in ICU survivors, in those survivors with low muscle mass, the pilot data revealed no difference in the satellite cell content of trained versus untrained muscle, suggesting that impaired muscle regenerative capacity may contribute to persistent muscle wastage post ICU discharge. This pilot project serves to inform the use of BFRE in large-scale trials to determine the role of the satellite cell and molecular regulation of sustained muscle wasting and weakness in critical illness survivors.

## Materials and methods

### Study participants

Critical illness survivors were recruited from previous participants in the RECOVER study at St Michaels Hospital (SMH) Unity Health Toronto (Toronto, Canada) who had consented to future contact for involvement in additional research studies. RECOVER was a Canadian multi-center prospective longitudinal study to evaluate functional outcomes in critically ill patients (age 16 years or older) following prolonged mechanical ventilation (1 week duration or more) over a 1 year period after ICU discharge (Herridge et al., 2016). Exclusion criteria were current or previously documented neurological injury that could preclude completion of questionnaires, formal documentation of neuromuscular disease, non-ambulatory before critical illness, documented history of psychiatric illness or significant cognitive impairment, and not fluent in English.

All patients who survived to hospital discharge and were living within the city of Toronto, Canada and surrounding area (radius 200 km) at the time of participation in RECOVER were sent a postal invitation to participate in the current study. The restriction on place of residence was established due to the necessity for daily on-site, supervised study participation. If there was no reply to the invitation, a second mailing was sent 4 weeks later. Survivors were excluded if they could not commit to attending the exercise training program daily or were on systemic corticosteroid or therapeutic anticoagulation. Muscle biopsy was not permitted solely for study purposes in anticoagulated survivors by the Research Ethics Boards (REBs). Healthy control individuals were recruited using posters placed at the University of Toronto Rehabilitation Sciences Building, University of Toronto (Toronto, Canada). Informed consent was obtained from all study participants. The study was approved by the Research Ethics Boards of Unity Health Toronto (REB 16–351) and the University of Toronto (REB #34268).

### Study protocol

Baseline demographics included age, sex, height in meters, and weight in kilograms (on a standing scale). Quadriceps muscle was selected for testing because of ease and validity of measures of size, strength, and suitability for percutaneous muscle biopsy under local anesthetic. The dominant leg was self-reported for each subject as the leg they used to kick a ball. The dominant limb was designated the exercise trained limb and the non-dominant was the control untrained limb. Subjects underwent baseline testing to determine rectus femoris (RF) and vastus lateralis (VL) muscle size using ultrasound and knee extensor muscle strength using a computerized dynamometer. This was followed by quadriceps BFRE training of the dominant leg, with the non-dominant leg not exercised. Following the exercise training session on the last day (Day 5), subjects repeated the quadriceps strength and ultrasound measures, as well as a quadriceps cross-sectional CT scan and biopsy of the VL muscles of both the exercise-trained and -untrained control limb. The muscle biopsies were performed last, between 3 and 5 h following completion of the final exercise session of the BFRE regimen.

### Novel low-intensity, short-course BFRE regimen

The novel BFRE regimen design was based on a thorough review of published regimens (e.g., Nielsen et al., 2012; Wernbom et al., 2013; Sieljacks et al., 2019) with the aim of both minimizing exercise intensity and duration and yet retaining the capacity to stimulate satellite cell proliferation and induce differences in transcript levels of mediators of muscle proteolysis, autophagy, and myogenesis in healthy muscle commensurate with those observed for high intensity resistance exercise (Sebastian and Zorzano, 2020; Yang et al., 2005; Yang et al., 2006; Louis et al., 2007; Telles et al., 2023).

At baseline, each subject performed maximum voluntary isometric contractions (MVIC) of the knee extensors on both their dominant and non-dominant limbs using a Biodex isokinetic dynamometer (System 4 Pro, Biodex Medical Systems). Five MVICs were performed at 90 degrees of knee flexion, and the highest three values (within 10%) were averaged to determine the peak isometric torque (Kireilla et al., 2018).

Subjects underwent five daily supervised training sessions of the dominant leg on the Biodex dynamometer. The training load was set at 30% of the peak isometric torque, and training was done in the isotonic mode. To restrict blood flow to the limb during exercise, a pneumatic cuff (15.0 cm width) was placed around the proximal portion of the thigh. The cuff was inflated to 140 mmHg, or 20% higher than the measured systolic blood pressure of the arm (whichever was the higher value). The participant performed a warm-up set of ten repetitions of isotonic knee extension at 30% of peak torque with no cuff inflation. The BFRE program consisted of eight sets of eight knee extensions, separated by a 30 s rest period between each set with the cuff inflated to the pre-determined pressure. During the rest period, the cuff pressure was deflated to 60 mmHg or completely deflated, as tolerated by the participant. The exercise volume (sets *x* repetitions), peak velocities (deg/second), and work performed (Joules) were recorded for each daily exercise session. On day 5, following the final training set, subjects were given a 30-min rest, and the isometric voluntary peak torque was again determined for the trained and untrained quadriceps.

### Muscle soreness and effort assessment

Immediately after each training session, subjects were asked to rate the level of fatigue in their exercised muscle using validated measures including a rate of perceived exertion (RPE) scale and to rate the sensation of delayed onset muscle soreness (DOMS) in their legs as they moved slowly from standing to sitting in a chair (i.e., during an eccentric contraction of the quadriceps) (Mathur et al., 2005; Cleather and Guthrie, 2007; Lau et al., 2015; Jo and Bilodeau, 2021). Their DOMS rating was marked on a 10-cm visual analogue scale (VAS) from “no soreness” (0 cm) to “maximal soreness” (10 cm). Subjects used a body diagram to shade areas where they felt DOMS. A simple qualitative description of sensation was also obtained from the participant every day after training.

### Quadriceps ultrasound imaging

B-mode ultrasound (General Electric Logiq *e*; GE Medical Systems, Milwaukee, WI) of the quadriceps muscle was performed by a trained operator to obtain muscle thickness and cross-sectional area of the RF and muscle thickness of the VL muscles at 50% of thigh length (from ASIS to superior pole of patella). The ultrasound frequency was set at 12 MHz, with gain (58–72 dB) and depth (4–6 cm) settings optimized by the operator to obtain a clear image of the muscle. Captured images were exported from the ultrasound system in DICOM format to Osirix MD software (https://www.osirix-viewer.com/osirix/osirix-md/) and manually outlined to obtain measures of muscle thickness or cross-sectional area, as per Mendes et al. (2015) and Kwan et al, (2020). The average measurement from three images was used for statistical analysis.

### CT imaging of the Mid-thigh

Approximately 2–4 h following the final BFRE training session, CT scan of the right and left thighs simultaneously on cross section, as described by Plant et al. (2010), was performed. Briefly, CT imaging of the thighs, halfway between the pubic symphysis and the inferior condyle of the femur, was completed with the study subject in the supine position. Each image was a maximum of 20 mm thick, and the muscle was identified as a tissue with a density of 40–100 Hounsfield units. Images were analyzed to determine the CSA of the thigh muscles by a single radiologist who was blinded as to the categorization of each subject and exercise trained versus the untrained control limb.

### Muscle biopsy

Percutaneous biopsy of the VL of both right and left thighs was performed approximately 3–5 h following the last training session on Day 5 (see dos Santos et al., 2016). Briefly, under sterile conditions, the skin and subcutaneous tissue were anesthetized, and a small incision was made in the outer fascial layer with a scalpel blade at the upper portion of the distal third of the muscle. A Bergstrom needle was advanced through the incision roughly 1 cm into the muscle, and suction applied as the trochar was advanced, to obtain approximately 200 mg of muscle in a single pass. The incision was closed with a single suture and removed 5 days later. The muscle was flash-frozen in isopentane for histologic assays and in liquid nitrogen for RNA. Biopsies were stored at −80C prior to experimentation.

### Immunohistochemistry

Frozen histologic sections cut on cross section (7 um) were fixed (4% paraformaldehyde) for 10 min, washed in phosphate buffered saline (PBS), permeabilized (0.1% Triton, 0.1M glycine in PBS) for 10 min, and blocked (2% BSA, 5% FBS, 5% goat serum, 0.2% Triton, and 0.1% Na Azide in PBS) for 90 min. Sections were incubated with Pax7 antibody (Developmental Hybridoma Bank, 1:2 dilution) overnight at 4 ℃, washed with 0.05% tween in PBS, and incubated with Alexa Fluor-555 secondary (1:500 dilution, Invitrogen A21428) for 1 h at room temperature (RT). Pax 7 is an exclusive marker of satellite cells in muscle histologic sections (Zammit et al., 2006). Slides were washed, re-blocked (10% goat serum) for 1 h, incubated for 1 h at RT with laminin antibody (1:500 dilution, Sigma L9393) or fast myosin skeletal heavy chain antibody (MY-32 1:800 dilution Sigma M4276), and washed and incubated with Alexa Fluor-488 (1:500 dilution, Invitrogen A11008) x 1 h RT. The anti-fast myosin antibody specifically targets the fast-twitch/type 2 isomyosin in skeletal muscle, staining type 2 myofibers but leaving slow twitch type 1 myofibers unstained. Slides were washed (tween 0.05% in PBS), nuclei labeled with Hoechst (1:10,000) for 3 min, washed with PBS, and cover-slipped with Dako fluorescence mounting media. Sections incubated with Hoechst and secondary antibodies only (Alexa-Fluor-488 and-555) served as negative controls.

### Myofiber morphometrics and Pax7 content

Whole tissue sections were scanned with the Zeiss Axioscan.Z1 (20x/0.8PlanApochromat, Hamamatsu ORCA-Flash 4.0 camera, Zen 2.6 software). Slides were manually reviewed by individuals blinded to histological sections to determine myofiber type-specific CSA, nuclear number, and the number of Pax7 positive cells/myofiber using Fiji v1.58 and Halov 2.3.2089 software. Myofibers were identified and determined to be on cross-section if size ranged between 25 and 22,000 square microns and fiber circularity fell between 0.50 and 1. Section fields for Pax7 quantitation were delineated that contained, at minimum, a total of 100 myofibers cut on cross section, and Pax7+ve (satellite) cells within those section fields were counted by the blinded observer. The total number of satellite cells was divided by the total number of myofibers to delineate Pax7+ve cells/myofiber. Pax7 +ve cells were also counted in section fields of myofibers oriented on cross section and expressed as Pax7+ve cells/μm^2^. Fold difference was determined as Pax7+ve cells/myofiber or μm^2^ in the BFRE-trained limb divided by Pax7+ve cells/myofiber or μm^2^ in the untrained limb, respectively.

### RNA extraction and droplet-Digital PCR (ddPCR)

Muscle tissue snap-frozen in liquid nitrogen was homogenized in TRIzol (1 mL/mg tissue; Life Technologies, Burlington, ON, Canada) and processed using a commercially available RNA Extraction Kit (Qiagen, 74,004, Germantown, MD, United States) as per the manufacturer’s instructions to isolate total muscle RNA. Briefly, muscle was placed in liquid nitrogen in an RNAse free mortar and pestle, crushed into powder and transferred to a 5-mL round bottomed tube on ice containing TRIzol. Samples were homogenized in three 30-s intervals (Kinematica AG, Lucern, Switzerland) and then processed and DNAse treated on column as per the manufacturer’s instructions. cDNA was then generated from a total of 65 ng of DNAse free RNA in a first-strand synthesis reaction using Superscript III First- Strand Synthesis SuperMix (Invitrogen, Burlington, ON, Canada).

Droplet Digital (ddPCR) was used to assess the expression of MuRF1, atrogin-1, myostatin, Beclin-1, LC3, myogenin, and Myf5 mRNA in VL biopsies from BFRE-trained and untrained legs. Primers were previously designed using Primer Express software (Applied Biosystems; Plant et al., 2010) and validated. Primer sequences are shown in Supplementary Table S1. We loaded 20 ul reaction mixtures per well consisting of 11 ul QX200 ddPCR EvaGreen Supermix (Biorad, 1864034), forward and reverse primers (150 nM), cDNA (200 pg), and remaining (up to 20 ul) volume nuclease-free water in a 96-well plate (#12001925 10 Bio-Rad) in the AutoDG Droplet Generator (Biorad). Following droplet generation, the plate was sealed with a foil heat seal (#181–4040, Bio-Rad) using the PX1 PCR Plate Sealer (#181-4000, Bio-Rad) and loaded into the PCR thermocycler (C1000, Biorad). PCR reaction was initiated with a 95 °C enzyme activation step for 5 min, followed by 40 amplification cycles (94 °C for 30 s and 60 °C for 1 min per cycle), a final extension step for 10 min, and subsequently left at 4 °C. Post-cycling droplet detection and absolute quantification of mRNA copies/200 pg cDNA were performed with the QX200 Droplet Reader (Biorad); data was processed and analyzed using Quantasoft (v.1.7.4) software.

### Statistical analysis

Statistical analysis was completed with GraphPad Prism (10.2.3). For the healthy control study participants, one-way ANOVA and Sidak’s post-hoc multiple comparison test assessed all measures completed in the untrained limb, and pre- and post-BFRE in the trained limb. Paired t-tests were used to compare measures completed in only the untrained limb and BFRE post-trained limb. A Shapiro–Wilks test was used to test for normal distribution. Pearson correlation co-efficient was determined for the relationship between total work done and fold difference in muscle Pax7+ve cells in trained versus untrained VL. Statistical significance was set at p < 0.05. While the critical illness survivor cohort enabled descriptive assessment of BFRE tolerability, power analysis revealed N to be inadequate for statistically evaluating satellite cell differences and muscle response in the cohort.

## Results

### Study participants

Of the 85 RECOVER patient participants enrolled at SMH, 23 had originally consented to being contacted for future studies. Invitations for participation in the current study mailed to 6 of 23 patients could not be delivered (returned to sender), no response was received to 12 invitations, and 5 patients, now designated as “critical illness survivors”, responded affirmatively to the invitation. Two of the five survivors were on therapeutic anticoagulation, precluding muscle biopsy and were thus excluded. Three survivors consented to study inclusion (2 males, 1 female); all were 5 years or more post ICU discharge.

ICU admission diagnoses and lengths of stay (LOS) for male survivors A and B were sepsis/acute kidney injury (41 days LOS) and thoracic cage/abdominal trauma (17 days LOS) respectively, and pulmonary renal syndrome for female survivor C (88 days LOS). At the time of participation in RECOVER, survivor C had no self-reported medical co-morbidities but was obese; survivor B self-reported hypertension; survivor A self-reported hypertension, hyperlipidemia, and prior pancreatitis. To determine participants’ pre-ICU exercise tolerance, the study protocol administered the Duke Activity Status Index (DASI) questionnaire, which grades exercise tolerance and cardio-respiratory reserve based on the individual’s self-reported ability to undertake various levels of activity (Hlatky et al., 1989). Participants’ physical functional capacity and global strength 7 days post-ICU discharge were determined using the Functional Independence Measures (FIM) motor subscore, which grades physical functional status from total independence (maximum score 91) to total assistance (Oczkowski and Barreca, 1993), and the MRC sum score, which manually grades muscle strength (maximum score 60, indicating full strength appropriate for age and sex; Hermans et al., 2012; Kleyweg et al., 1991), respectively. Survivors A, B, and C all self-reported the ability to undertake strenuous physical activity prior to ICU admission, as indicated by obtaining the maximum score (58.2) on the DASI questionnaire. All survivors developed ICUAW during their critical illness, and all demonstrated persistent weakness and physical functional limitations at 7 days post ICU-discharge. The FIM motor subscore and MRC sum score for male survivor A were 50 and 48 respectively, and 55 and 50 respectively for male survivor B. The 7-day post-ICU discharge FIM motor subscore and MRC sum score were 26 and 26 respectively for female survivor C.

At the time of consent to the current study, all critical illness survivors self-reported no change in medical co-morbidities since ICU discharge, were not on myotoxic drugs (i.e., corticosteroid), were ambulatory without aids, and were non-sedentary.

Eight healthy active individuals (five men and three women) consented to study participation as control participants.

### BFRE training program metrics

**FIGURE 1:**
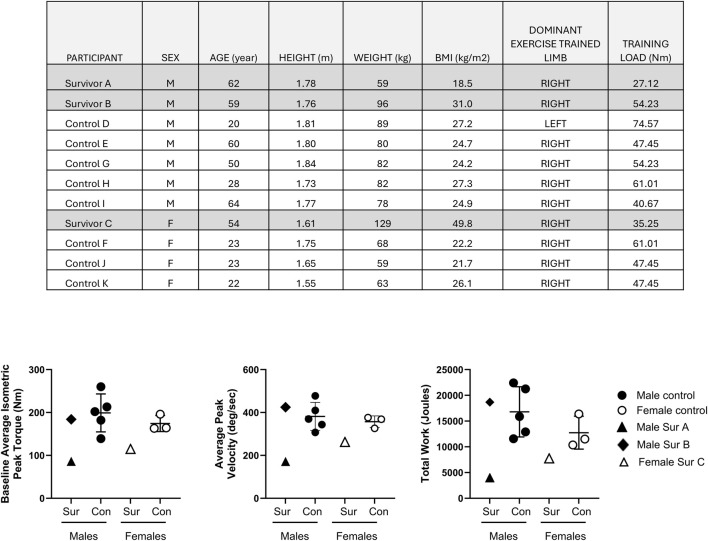
Table l shows participant demographics, dominant limb and training load use for the BFRE regimen. Graphs depict the baseline quadriceps isometric peak torque of the dominant limb (left panel), average peak velocity (middle panel) and total work performed (right panel) over the 5 day BFRE program. Critical illness survivors A and C demonstrated lower baseline quadriceps peak torque, average peak training velocities and total work performed compared to sex-matched controls. The baseline quadriceps peak torque, average training velocity and total work performed for survivor B fell within the observed values for sex-matched controls. (Control participant data are mean +/– SD; Sur = survivor, Con = control).

All study participants undertook the five day supervised BFRE regimen on a Biodex dynamometer of their dominant leg quadriceps. Subject demographics, baseline quadriceps isometric peak torque, training load, and total work completed during the 5‐day training period are shown in Figure 1. Survivors A and C demonstrated the lowest baseline quadriceps peak torque at study initiation and average peak velocities during BFRE, and they performed less total work over the 5-day BFRE program than the healthy control participants and survivor B (Figure 1).

### BFRE tolerability

All critical illness survivors and control study participants completed the entire 5-day BFRE training program. All critical illness survivors and control participants indicated fatigue of the trained leg during testing, with RPE scores ranging from minimal (0.5) to maximum (10) fatigue (Table 1). All critical illness survivors and the majority of healthy participants (88%) also reported negative qualitative muscle sensations such as burning, aching, tightness, and numbness during BFRE. In contrast, the majority of study participants reported no DOMS.

**TABLE 1:** Metrics of leg fatigue and discomfort were obtained each day after BFRE training using the Rated Perceived Exertion Scale [RPE] and delayed onset muscle soreness (DOMs) visual analog score (VAS) respectively. The RPE and DOMs VAS scores range from 0 (none) to 10 (maximal) leg fatigue and soreness, respectively. Self-reported qualitative leg sensations were also recorded daily. (NR = not recorded).

Participant	RPE scale leg fatigue		Leg sensation at end of exercise					DOMS VAS scale (CM)	
	Day 1	Day 5	Day 1	Day 2	Day 3	Day 4	Day 5	Day 1	Day 5
Survivor A	10	10	Aching	Fatigue	Tired, Burning	Burning	Burning	0.8	0.2
Survivor B	9	5	Burning	Burning	Stiff	Burning	Burning	0	4
Survivor C	9	2	NR	None	Tingling	Tingling	Heavy	0	0
Control D	4	10	Numb	Sore	Sore	Sore, Numb	Sore	0	2.8
Control E	0.5	7	Sore	Sore	Sore	NR	Fatigued	0	0
Control F	8	9	NR	NR	NR	NR	NR	0	0
Control G	5	4	Sore	Tightness	Tightness	Tightness	Sore	0	0
Control H	9	10	Tightness	Burning	Burning	Tightness	Burning	0	0
Control I	10	10	Sore	Burning	Sore	Sore	Most Sore	4.6	2.4
Control J	7	7	Tightness	Tightness	Tightness	Tightness	Tightness	0	0
Control K	6	3	Very Sore	Very Sore	Very Sore	Very Sore	Pain	2	0

### Quadriceps size

**FIGURE 2:**
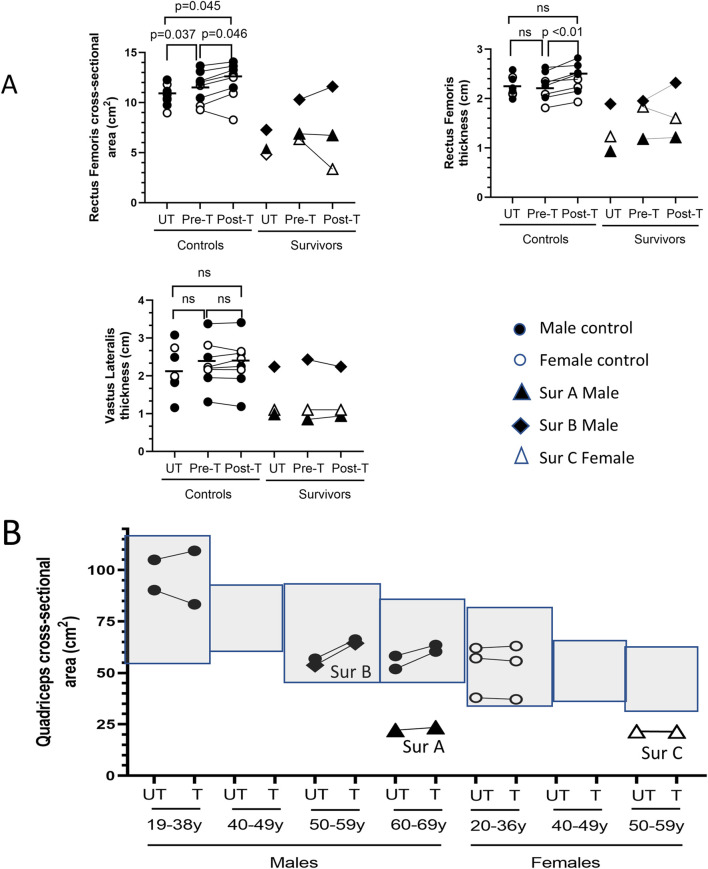
BFRE impact on quadriceps size. **(A)** Ultrasound determined rectus femoris (RF) cross-sectional area (CSA) and thickness but not vastus lateralis (VL) thickness, increased post-BFRE training in control subjects. RF and VL metrics for critical illness survivor B fell within the range of values for control participants. Survivors A and C RF and VL muscles were smaller. **(B)** Light grey boxes represent published sex- and age-specific quadriceps CSA (mean +/- 95% confidence intervals) of healthy individuals (Dos Santos et al., 2016; Kasai et al., 2015; Young et al., 1985; Schantz et al., 1983; Segal et al., 2011; Maughan et al., 1983; Housh et al., 1995; Hakkinen et al., 1993; Kanehisa et al., 1994; Goodpaster et al., 2001). CT determined BFRE-trained and -untrained quadriceps CSA of control subjects (circles) fell within the published age- and sex-matched norms, and the CSA of the trained versus untrained quadriceps was not different. Quadriceps CSA of survivor B but not survivors A or C fell within the population-based age- and sex-matched norms. (n.s. = not significant [p ≥ 0.05]; T = trained leg; UT = untrained leg; Sur = critical illness survivor; - data mean).

Prior to BFRE training, the baseline rectus femoris (RF) cross-sectional area (CSA) of the dominant leg was slightly larger than the non-dominant leg (11.51 ± 1.58 vs. 10.67 ± 1.07, p = 0.037) in control participants, but there was no difference in the baseline RF or vastus lateralis (VL) thickness (Figure 2A). The 5-day BFRE regimen induced a small increase in RF CSA (pre-BFRE 11.51 ± 1.58 cm^2^ vs. post-BFRE 12.12 ± 1.88 cm^2^, p = 0.046) and thickness (pre-BFRE 2.25 ± 0.27 cm vs. post-BFRE 2.40 ± 0.29 cm, p < 0.01) of the trained leg in control participants as determined by ultrasound, but did not increase vastus lateralis (VL) thickness. Similarly, there was no difference in the whole quadriceps CSA of the BFRE trained versus untrained legs in control participants as determined by CT scan (67.35 ± 21.20 cm^2^ vs. 64.92 ± 21.77 cm^2^ respectively, p = 0.25). Additionally, the muscle CSA for both legs fell within published population-based sex- and age-matched norms for healthy individuals (Figure 2B; Dos Santos et al., 2016; Kasai et al., 2015; Young et al., 1985; Schantz et al., 1983; Segal et al., 2011; Maughan et al., 1983; Housh et al., 1995; Hakkinen et al., 1993; Kanehisa et al., 1994; Goodpaster et al., 2001).

The quadriceps CSA for critical illness survivors A and C, however, fell below the published sex- and age-matched norms for healthy individuals (Figure 2B), and the ultrasound determined measures of RF and VL size were similarly below the range of metrics for the control study participants (Figure 2A). In contrast, the quadriceps CSA of survivor B fell within the published sex- and age-matched norms for healthy individuals (Figure 2B). The RF and VL CSA and thickness for survivor B also fell within the range of the study control participants (Figure 2A).

**FIGURE 3:**
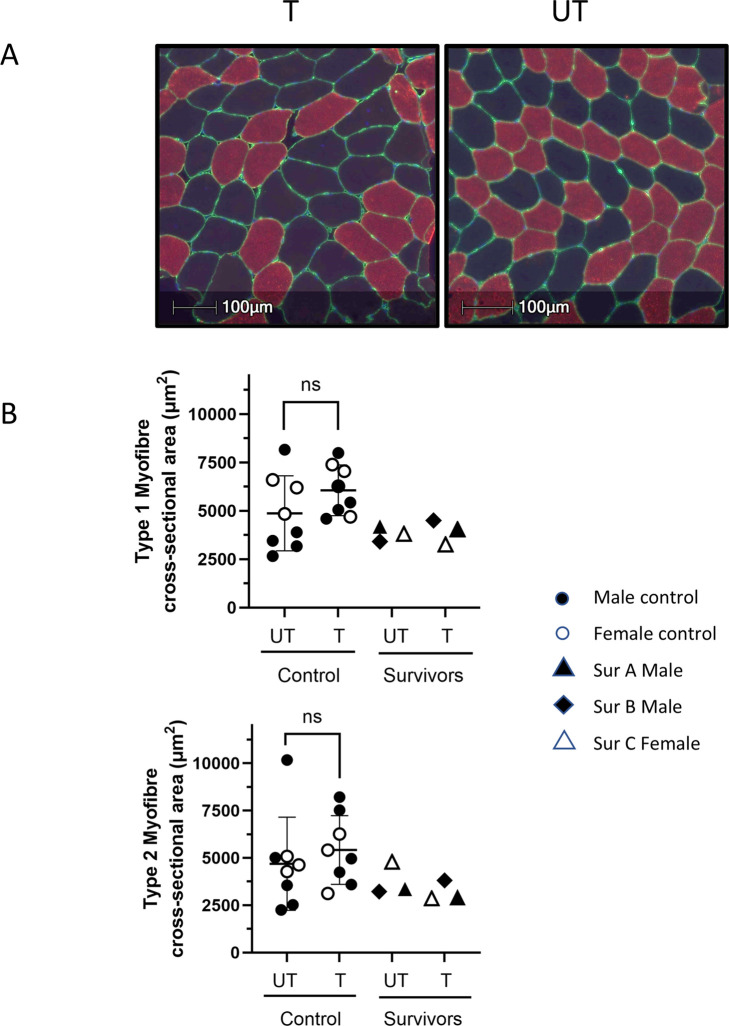
BFRE impact on myofiber cross-sectional area (CSA). **(A)** Representative histological cross-sections of vastus lateralis (VL) biopsies from BFRE-trained and untrained legs immunostained for type 2 myosin heavy chain (red). Type 1 myofibers are black. Laminin immunostaining (green) indicates the endomysium. Hoechst indicates nuclei (blue). **(B)** Morphometric analyses did not demonstrate differences in type 1 or 2 myofiber CSA in the trained versus untrained muscle in control or survivor participants. (T = trained, UT = untrained, ns = not significant [p ≥ 0.05], Sur = critical illness survivor; data are mean +/-SD).

To assess the impact of BFRE on myofiber CSA, study participants underwent biopsy of the vastus lateralis of both the trained and untrained legs 3–5 h after the final BFRE session (Figure 3). No differences in CSA were observed for control participants, and the myofiber cross sectional area of all three survivors fell within the range of size for the control participants.

### Quadriceps strength

**FIGURE 4:**
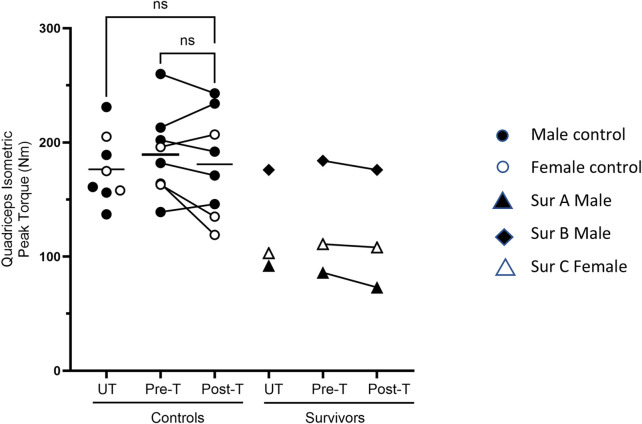
Quadriceps strength post-BFRE. BFRE training did not significantly increase the quadriceps isometric peak torque. The baseline peak torque of critical illness survivor B, but not survivors A or C, fell within the range of the control participants (ns = not significant [p ≥ 0.05], T = trained, UT= untrained, Sur = critical illness survivor, - data mean).

The 5-day BFRE training regime did not increase the quadriceps’ isometric peak torque (Figure 4).

### Vastus lateralis transcript expression of the regulators of proteolysis, muscle mass, autophagy and myogenesis

**FIGURE 5:**
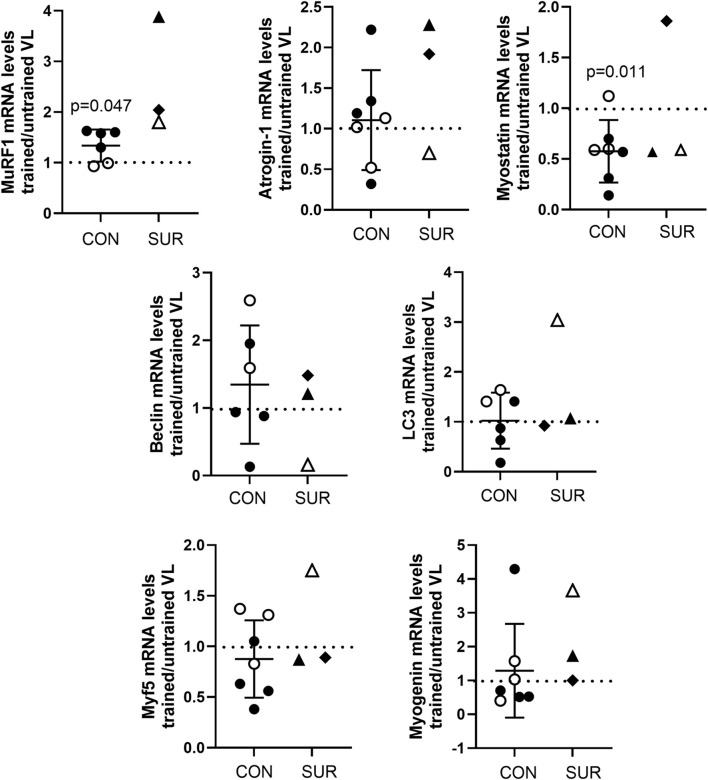
Fold difference in transcript expression levels as determined by ddPCR for proteins regulating muscle proteolysis and atrophy (MuRF1, atrogin-1, and myostatin), autophagy (Beclin-1 and LC3), and myogenesis (Myf5 and myogenin) in vastus lateralis (VL) of BFRE trained versus untrained legs 3 to 5 hours post-final training session. MuRF1 was significantly higher and myostatin significantly lower in the BFRE-trained versus untrained VL of control participants (CON = control participants, SUR = critical illness survivor, data are mean +/– SD, dotted line indicates level of no difference in transcript expression).

RNA was extracted from the biopsies of the trained and untrained VL to determine transcript expression. Inadequate RNA was available for one female control participant for evaluation. In control participants, transcript expression of the ubiquitin ligase MuRF1 was significantly higher in the BFRE trained versus untrained VL, while myostatin transcript levels were significantly lower (Figure 5). The expression level for the majority of transcripts in the survivors fell within a range similar to that observed for the control participants with the exception of MuRF1, where the fold difference in transcript expression in BFRE trained vs. untrained VL appeared to exceed that of the control participants (Figure 5).

### Vastus lateralis satellite cell content

**FIGURE 6:**
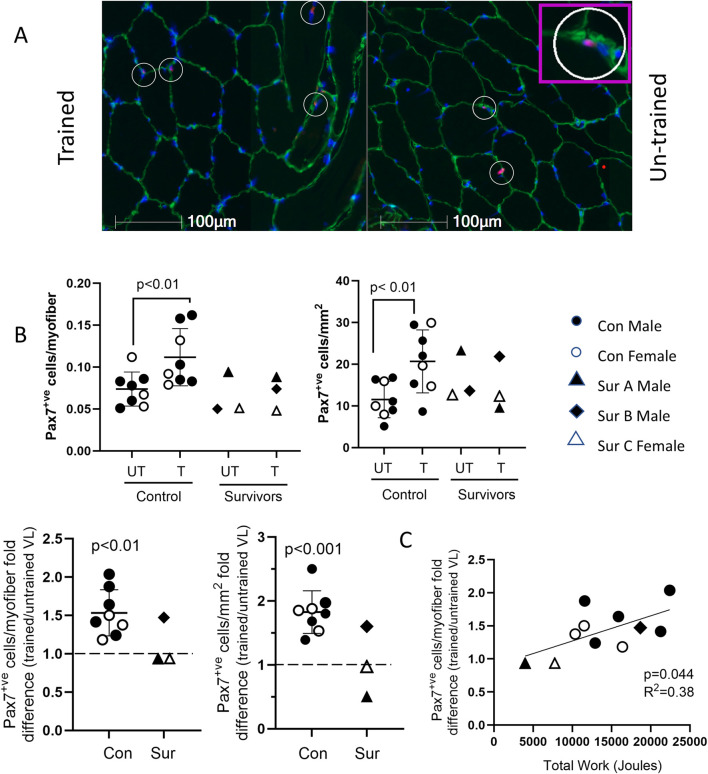
VL satellite cell content following 5-day BFRE program. **(A)** Representative images of vastus lateralis (VL) cross sections immunostained for Pax7 (red, satellite cell marker), laminin (green), and nuclei indicated with Hoechst (blue). Pax 7+ve cells appear pink and are indicated with circles (inset shows magnified image). **(B)** VL satellite cell content (shown as cells/myofiber and cells/mm^2^) was higher in BFRE-trained versus untrained leg in control participants and survivor B but not survivors A and C (control data are mean+/-S.D; dotted line represents no fold difference). **(C)** Fold difference in VL satellite cell content of BFRE trained versus untrained muscle correlated significantly with the total work completed over the 5-day program. (Con=control, Sur = critical illness survivor).

In control participants, the VL satellite cell content was significantly higher in the BFRE-trained vs. -untrained leg (Figures 6A, B). Stratifying the control population by age, participants both less than and greater than 30 years demonstrated significantly higher satellite cell content in the BFRE trained vs. untrained VL. Mean fold differences in satellite cell count/myofiber and count/mm^2^ were 1.50 ± 0.32 (p = 0.02) and 1.91 ± 0.37 (p < 0.01), respectively, in control participants under 30 years, while participants over 30 years demonstrated mean fold differences of 1.56 ± 0.27 (p = 0.03) and 1.84 ± 0.06 (p = 0.02), respectively.

As with the control participants, satellite cell content was higher in the BFRE trained versus untrained leg for survivor B (Figure 6B). In contrast, for survivors A and C there was no apparent difference in the satellite cell content of the trained versus untrained VL. For all study participants, there was a significant correlation between total work completed over the 5‐day BFRE training program and the fold difference in myofiber satellite cell content of the BFRE-trained VL (Figure 6C).

## Discussion

In this study, we show that a novel BFRE regimen, purposefully designed to be of minimal intensity and duration, was successfully completed by all healthy control and critical illness survivor study participants. Importantly, a higher VL satellite cell content and differences in transcript levels of molecular regulators of proteolysis and muscle mass were observed in the BFRE-trained vs. -untrained leg of the healthy control participants consistent with those previously reported for resistance exercise programs of higher intensity and/or longer duration (Yang et al., 2005; Yang et al., 2006; Louis et al., 2007; Telles et al., 2023; Nyakayiru et al., 2019; Manini et al., 2011; Drummond et al., 2008; Laurentino et al., 2012; Fritzen et al., 2016). This study supports the use of a short-course, low intensity BRFE regimen as a feasible study intervention for investigating the molecular mechanisms and satellite cell role in regulating the failure vs successful recovery of muscle mass and strength in critical illness survivors following ICUAW.

Study of the mechanisms underpinning the impaired recovery of muscle mass and strength following critical illness currently requires translational studies in humans. While pre-clinical models of ICUAW exist, they involve continual sedation with or without paralysis of the animal during mechanical ventilation for days (porcine) or weeks (rodent) (Banduseela et al., 2009; Ochala et al., 2011a; Ochala et al., 2011b; Norman et al., 2006; Ackermann et al., 2014). The clinical standard of care to minimize and expediently discontinue sedation and paralytics in mechanically ventilated humans is not possible in animals, so the ICUAW inciting events at baseline are, by necessity, different. Moreover, animal survival post mechanical ventilation to evaluate long-term outcomes and muscle sequelae can be very difficult to achieve.

Muscle biopsies following resistance training in humans have enabled study of the molecular mechanisms that regulate muscle mass and strength. High intensity resistance training at loads greater than 60% of maximum strength (1-repetiiton maximum; 1-RM) is well documented to stimulate satellite cell activation, proliferation, differentiation and incorporation into new and existing myofibers to increase muscle mass. However, the response of the satellite cell to resistance training with BFRE is more variable. While a 33%–53% increase in quadriceps satellite cell numbers was reported after a single BFRE session of five sets of knee extension repetitions to failure at 30% 1-RM in young healthy participants (Wernbom et al., 2013), the vast majority of studies evaluating BFRE programs did not take repetitions to the extreme of muscle failure and they significantly extended the training duration (8 days or more). The reported satellite cell response ranges from no impact to 296% increase from baseline numbers (Nielsen et al., 2012; Wernbom et al., 2013; Sieljacks et al., 2019; Bjorsen et al., 2021). The reasons for the different outcomes observed are not clear but likely result from a combination of varied factors between studies, including duration and intensity of exercise, sex, age, the muscle(s) trained and timing of biopsy following BFRE training.

The intensity and duration of training become key issues to address when the goal is to study the satellite cell in a cohort of individuals who may be older with significant medical co-morbidity or frailty, such as ICU survivors, which can prevent completion of the cumulative muscle work prescribed. Moreover, 67% of primary caregivers of critical illness survivors have been shown to suffer from burn-out and depression (Cameron et al., 2016), which may inhibit their ability to transport and accompany the dependent survivor to a research study session. Thus, an exercise program of minimal muscle effort and time commitment that accommodates the potential limitations of both survivor and caregiver will best ensure that the most vulnerable individuals have the opportunity to be included in research, thus minimizing bias.

In this study, the two survivors with low quadriceps muscle mass, when compared to both population-based sex- and age- matched norms and the study control cohort, were also the weakest study participants at baseline. They thus achieved the lowest levels of total work among all study subjects due to their lower training loads. Given that we observed a positive correlation between total muscle work performed and VL satellite cell content, is not clear whether the apparent absence of a BFRE impact on the muscle satellite cell content in these survivors is due to the lower absolute amount of work performed not reaching an adequate stimulatory threshold, or is the result of some durable impairment of the satellite cell proliferation that persists following ICUAW. This will require further study and validation in a larger ICU survivor cohort. Furthermore, while baseline measurements for the survivors’ quadriceps size at ICU admission were not available, the self-reported ability for all to undertake strenuous exercise prior to their critical illness suggests that their pre-ICU muscle mass might have fallen within “normal” limits. Thus, the low quadriceps mass observed in two of the survivors may indicate persistent muscle wasting post ICU discharge. It can be conjectured that the absence of a satellite cell response to the extent of muscle work they were able to perform could contribute to the persistent muscle wasting observed years after critical illness resolution. In keeping with this postulate, the survivor who had “normal” quadriceps mass years post ICU discharge, based on comparable metrics to population norms and the study control cohort, completed total work in the BFRE program and had a positive fold difference in satellite cell content with BFRE similar to controls, suggesting an ability to regenerate muscle mass.

MuRF1 and atrogin-1 are both ubiquitin ligases that are positive regulators of the ubiquitin proteasome system (UPS), inducing muscle proteolysis and atrophy with sustained increases in expression (Peris-Moreno et al., 2021). However, MuRF1 mRNA levels have also been reported to increase rapidly and stay elevated for about 5 h following high load and BFRE as part of an acute catabolic response to rid the cell of damaged protein prior to falling below baseline levels 8–12 h after exercise stimulus (Yang et al., 2006; Louis et al., 2007; Nyakayiru et al., 2019; Manini et al., 2011; Drummond et al., 2008; Mascher et al., 2008). In contrast, atrogin-1 does not show a biphasic response post resistance exercise, and transcript levels are only reported to begin falling about 6 h following exercise stimulus (Yang et al., 2006; Louis et al., 2007; Nyakayiru et al., 2019; Manini et al., 2011; Drummond et al., 2008; Mascher et al., 2008). In keeping with the literature, at 3–5 h post exercise we found a significantly higher MuRF1 transcript expression in BFRE trained versus untrained muscle, with no differences noted in atrogin-1 mRNA levels, in control participants. Similarly, myostatin, which is a negative regulator of muscle growth known to exhibit a sustained decrease within 2 h of high intensity resistance exercise or BFRE (Yang et al., 2006; Louis et al., 2007; Drummond et al., 2008; Laurentino et al., 2012; Mascher et al., 2008), behaved in the current study as reported in the literature and was lower in the control participants’ BFRE-trained vs untrained muscle.

Beclin-1 and LC3 are markers of autophagy (Yang et al., 2016), a process of degradation and recycling of cellular components that helps to maintain muscle basal cellular homeostasis. While endurance exercise induces autophagy in skeletal muscle, the role of autophagy in resistance exercise is unclear, as reports vary (Fritzen et al., 2016; Fry et al., 2013). We showed no difference in expression of either LC3 or Beclin-1 in the BFRE-trained versus -untrained legs in control participants, in keeping with the theory that autophagy may not be essential for muscle adaptation to resistance exercise (Sebastian and Zorzano, 2020).

Myf5 and myogenin, myogenic regulatory factors that contribute to the determination and differentiation of the skeletal muscle during post-natal myogenesis, are key to regenerative muscle growth (Hernández-Hernández et al., 2017) and are increased in response to high-intensity resistance exercise and BFRE (Yang et al., 2005; Telles et al., 2023; Drummond et al., 2008; Layne et al., 2016). However, the timeframe within which exercised induced changes in transcript expression occur is beyond the 3–5 h window evaluated in this study, and thus no differences in Myf5 or myogenin mRNA expression were observed in the control participants.

Transcript expression levels for the regulators of muscle mass in the critical illness survivors were mostly within the range observed for the control participants, with the exception of MuRF1, which was higher in the BFRE-trained leg of all three post-ICU survivors. This may suggest that the muscle of critical illness survivors is more prone to an exercise-induced catabolic response, but this requires further study with a larger ICU survivor cohort, as does its potential clinical relevance to muscle mass recovery post ICU discharge.

It is important to note that there was no difference in the myofiber type specific or whole quadriceps cross sectional area in the BFRE trained versus untrained legs in the control participants. Longer duration BFRE training protocols (Nielsen et al., 2012; Bjornsen et al., 2021; Vinolo-Gil et al., 2022) appear to be required. In contrast, the RF did demonstrate a clinically small but statistically significant increase in CSA and thickness with BFRE training. This may have been due to edema from exercise, given that myofiber diameters were not impacted. Importantly, strength was also not increased with the 5-day regimen, in keeping with studies of BFRE in individuals with neurological disease, which have demonstrated the inadequacy of shorter duration training programs to increase strength (Vinolo-Gil et al., 2022). In fact, in some instances in this study, torque measures of the trained limb were decreased compared to baseline, which may have been a result of cumulative fatigue influencing the voluntary strength measure in the trained leg.

The study has limitations. Of the 82 original patients in RECOVER, only 23 consented to future contact for additional studies, of whom six could not be located. Of the remaining 17, only five replied affirmatively to recruitment letters and three consented, generating a small post-ICU survivor sample of varied critical illness diagnoses, ICU LOS, and co-morbidities. ICU LOS, in particular, influences post-ICU physical functional outcomes (Herridge et al., 2016). Furthermore, we did not have a fully representative age range within the control participants; notably, all female controls were within their third decade. There are sex-specific differences in satellite cell content in type 1 and 2 myofibres in young individuals (Horwath et al., 2021a), but whether this impacts the response to exercise in older adults in not clear. Although older age is known to negatively impact satellite cell responses to exercise and injury (Sousa-Victor et al., 2022), we did not find age to be a confounding factor in the control participants in this study, possibly due to the absence of a fully representative age range.

We did not perform muscle biopsies pre and post BFRE, and instead biopsied the VL of both the trained and untrained legs simultaneously for comparison. Ethical considerations in critical illness survivors limited biopsies to one time point following the BFRE regimen to accommodate potential survivor frailty and to avoid the possibility of a prior invasive procedure in a profoundly wasted muscle confounding the results of a sequential biospy. While we found the baseline CSA of the RF of the dominant trained leg to be slightly larger than the untrained leg at study initiation, this was not the case for the VL thickness. Other studies have similarly reported no or small differences in quadriceps size between dominant and non-dominant legs (Wenzel et al., 2025; Lanferdini et al., 2023; Denadai et al., 2016). Prior studies have also reported symmetry between human right and left VL myofiber type proportion and CSA, myonuclei/fiber, myonuclear domain and transcript expression (Tarnopolsky et al., 2007; Horwath et al., 2021b), validating the use of the contralateral non-exercised muscle as an appropriate control for the trained muscle.

Given the study design, we were not able to assess serial changes in satellite cell content in response to BFRE, but instead only observed the higher VL satellite cell content in the trained vs untrained leg at BFRE completion. In future studies, co-immunostaining for proliferation markers (e.g., Ki67) and Pax7 could enable the direct detection of BFRE mediated stimulation of satellite cell proliferation. Similarly, the study design prevented following serial changes in transcript expression of key components of cellular signaling networks that regulate muscle mass after exercise. As the change in expression of mediators of muscle proteolysis, protein synthesis, and myogenesis all follow different patterns within the first 24–48 h following exercise, our single 3–5 h time point specifically missed the anticipated upregulation of myogenic regulatory factors. Future studies will need to either complete sequential biopsies after exercise or alternatively focus selectively on one process and obtain muscle at the appropriate time.

With the BFRE training, only the RF muscle and not the VL muscle increased in size. This differential effect may result from the fact that VL contributes to both knee extension and helps stabilize hip abduction during gait, so that the vector of motion with BFRE may have been less optimal for the VL. It is possible that the differences seen in satellite cell content and transcript expression of molecular regulators of muscle mass in the BFRE trained vs untrained muscle would have been greater had the RF been biopsied instead of the VL muscle. Future studies might consider both VL and RF biopsy for assessment.

As previously noted, although the two ICU survivors with low quadriceps mass were able to complete the BFRE regimen, given the low total workload they achieved over the 5 days, it is possible that the absence of any difference in the satellite cell content of the trained versus untrained leg resulted from an inadequate training stimulus. This could be addressed in future study by supplementing the voluntary contraction with neuromuscular electrical stimulation, to see if the forced increase in muscle work performed is able to stimulate a proliferative response in the satellite cell above that achievable by effort alone. In addition, we did not determine frailty indices for the critical illness survivors, which may have addressed functional limitations beyond muscle mass and strength.

Finally, the single female post-ICU survivor who consented to this study was older than the female controls, had extreme obesity and thus was not representative of the general population of critical illness survivors. There is extensive data demonstrating that obesity in itself impacts muscle form and function. Obese individuals are reported to have larger muscle mass overall, but when normalized to body or muscle mass, contractile strength is impaired due to physical phenomenon (muscle lipid infiltration and altered muscle architecture) as well as biochemical anomalies (impaired contractile function) (Cava et al., 2017; Tomlinson, et al., 2016; Choi et al., 2016; Tallis et al., 2018; Lafortuna et al., 2014). While this critical illness survivor was significantly weaker than controls, her muscle mass was also smaller than age- and sex-matched population-based norms, contrary to the expected impact of obesity, which may have resulted from impaired satellite cell function and muscle regeneration post ICU discharge. Importantly however, despite her obesity, she was able to complete the entire BFRE training program, thus raising the possibility of its potential use as a rehabilitative tool even for the significantly obese.

In summary, we have demonstrated that a novel low-intensity, short-duration BFRE regimen achieved a higher satellite cell content and differences in MuRF1 and myostatin transcript expression in BFRE-trained versus -untrained VL of healthy control individuals in keeping with reported responses to high intensity and/or longer duration exercise regimens. We have also shown that the BFRE program was tolerated and completed by all critical illness survivor participants. The use of BFRE will enable the prospective study of the cellular and molecular mechanisms of sustained muscle wasting and weakness versus recovery long-term post-ICU care in human subjects, specifically addressing the potential causal role of the satellite cell, informing future therapeutics development.

## Data Availability

The raw data supporting the conclusions of this article will be made available by the authors, without undue reservation.
