# Supplementary material for: A novel short-course, low-intensity blood-flow-restricted exercise (BFRE) regimen to study satellite cell function in critical illness survivors with sustained muscle atrophy following intensive care unit-acquired weakness (ICUAW)
Source: Front Physiol. 2025 Jun 18;16:1553471. doi: 10.3389/fphys.2025.1553471 (PMC12214465; doi:10.3389/fphys.2025.1553471)
Supplement: Supplementary file 1 [file DataSheet1.pdf]

Gene	Forward Primer	Reverse Primer
MuRF1	5' TCC AGC AGA CAC TGA ACC AGA A 3'	5' TCC ATT TTG CAC CAA TGT AGA AA 3'
Atrogin 1	5' GCA CGT GCT CAG CGA AGA 3'	5' ATC TGC CGC TCG GAG AAG T 3'
Myostatin	5' TTG AGA CCC GTC GAG ACT CCT A 3'	5' TTC AGA GAT CGG ATT CCA GTA TAC C 3'
Beclin-1	5' AGG AAC TCA CAG CTC CAT TAC 3'	5' AAT GGC TCC TCT CCT GAG TT 3'
LC3	5' ATG TCA ACA TGA GCG AGT TGG T 3'	5' CTG GTT CAC CAG CAG GAA GAA 3'
Myogenin	5' GCT GTA TGA GAC ATC CCC CTA CTT 3'	5' CGT AGC CTG GTG GTT CGA A 3'
Myf5	5' AGG TCA ACC AGG CTT TCG AA 3'	5' GAT GTA GCG GAT GGC ATT CC 3'

Supplementary Table 1. Primer sequences for ddPCR.
